# Digital Analysis of Sit-to-Stand in Masters Athletes, Healthy Old People, and Young Adults Using a Depth Sensor

**DOI:** 10.3390/healthcare6010021

**Published:** 2018-03-02

**Authors:** Daniel Leightley, Moi Hoon Yap

**Affiliations:** 1 King’s Centre for Military Health Research, Institute of Psychiatry, Psychology & Neuroscience, King’s College London, London WC2R 2LS, UK; 2 School of Computing, Mathematics and Digital Technology, Manchester Metropolitan University, Manchester M15 6BH, UK; m.yap@mmu.ac.uk

**Keywords:** kinect, depth sensor, motion capture, sit-to-stand, automated assessment, short physical performance battery

## Abstract

The aim of this study was to compare the performance between young adults (*n* = 15), healthy old people (*n* = 10), and masters athletes (*n* = 15) using a depth sensor and automated digital assessment framework. Participants were asked to complete a clinically validated assessment of the sit-to-stand technique (five repetitions), which was recorded using a depth sensor. A feature encoding and evaluation framework to assess balance, core, and limb performance using time- and speed-related measurements was applied to markerless motion capture data. The associations between the measurements and participant groups were examined and used to evaluate the assessment framework suitability. The proposed framework could identify phases of sit-to-stand, stability, transition style, and performance between participant groups with a high degree of accuracy. In summary, we found that a depth sensor coupled with the proposed framework could identify performance subtleties between groups.

## 1. Introduction

There is a clear and advancing benefit to the development of digitised automated systems to evaluate human motion using depth sensor technology for use in the healthcare domain [1,2,3]. The general population is living longer; therefore, new and innovative means of quantifying and assessing a person’s physical health are needed to better allocate resources and target interventions. While many in the ageing population will remain healthy, active, and engaged into later life, some studies have shown that a minority of older adults suffer from frailty and musculoskeletal disorders [4]. Focusing on frailty, it is not a single disease, but a combination of the natural ageing processes during which neuromuscular systems decline, and the accumulation of medical conditions leaves a person vulnerable to illness, trips, or falls [5]. Further, older adults have unstable balance and motion stability compared with the young, and the amount of body sway increases with more challenging motions [2,6]. 

Although in-person clinical assessment is vital, there is a need to develop more efficient clinical approaches that are suitable for the Internet-of-Things, assistive living [7], and Cloud Computing era [5,8,9]. There are many limitations in current assessment processes. First, clinician-led assessments are dependent on the skills, experiences, and judgement of the individual clinician, and therefore may not always be objective. Second, clinical assessments are open to subjective bias and contain inter-/intra-variance between assessments. Third, the entire process can be time-consuming considering the person’s need to attend the appointment and undertake the assessment, and the need for clinics to arrange appointments and oversee the assessments. Fourth, people with physical mobility impairment increase their risk of further trauma by having to attend specialist clinics, so it would be preferable to undertake the assessment at home or a suitable location. Fifth, a person may exhibit different behavior because of the examination, which may alter the outcome and perceptions by the clinician.

Several studies have utilised depth sensor technology to analyse and quantify mobility to predict possible future declines in physical health [1,10,11]. Early identification could enable remedial clinician-led intervention to occur more quickly and thus improve patient outcomes [3,4,12]. Several attempts have been made to develop assessment systems to judge clinically relevant motions such as sit-to-stand, timed-up-and-go, and static balance [13,14,15]. While these systems have been shown to be useful in monitoring and quantifying balance, they fall short of assessing time- and speed-related measurements between distinct population groups which could be insightful to a clinician in the decision-making process. 

There are several methods which seek to characterise sit-to-stand by decomposing the motion into phases to identify the start, middle, and end phase, and how the movement was performed [2,16,17]. Bennett et al. (2014) [16] used pressure sensors to gather movement data. The Centre of Mass was calculated and evaluated using a classifier to determine if different phases of motion could be identified. The authors could identify between slow, unstable sit-to-stand, and healthy transition phases. Ejupi et al. (2015) [13] used a depth sensor to examine the feasibility of detecting sit-to-stand motion between the elderly who may be prone to falling. By developing a system which uses time-and speed-related measurements, the authors could discriminate between those who were at high risk of falling and those who were not. 

In this paper, we propose a non-invasive markerless digitalised and automated framework, using novel feature generation and motion decomposition, to analyse the performance of masters athletes, healthy old people, and young adults performing the sit-to-stand (five repetitions) motion, which is a functional test that is commonly used in a clinical setting to assess balance and stability [18]. The framework acquires motion capture (mocap) data from a single depth sensor, where the skeletal stream is de-noised using a heuristic algorithm, then decomposed into a set of novel time-and speed-related features. Analysis techniques are employed to identify the performance in execution, sitting, and stand-to-sitting, thus providing detailed insight into the stages of motion analysis for clinicians. 

## 2. Materials and Methods 

### 2.1. Data Collection

A comprehensive description of the data collection for the K3Da dataset has been previously reported in [19], but a summary is provided hereafter.

#### 2.1.1. Participants

This study used the K3Da dataset [19], which consists of participants performing a range of clinically validated motions extracted from the Short Physical Performance Battery [18] under the supervision of a clinically trained individual and recorded using a markerless Microsoft Kinect One depth sensor (i.e., no devices are required to be affixed to the participant). Participants were recruited from staff at the Manchester Metropolitan University, a local athletics club, and elderly people from the general population. All reported that they had no history of neurological disorders or serious musculoskeletal injury, and each reported good upper and lower limb function. 

The data collected was approved by the local ethics committee of the Manchester Metropolitan University (SE121308). Written informed consent was obtained for each participant.

#### 2.1.2. Data Acquisition

**Figure 1:**
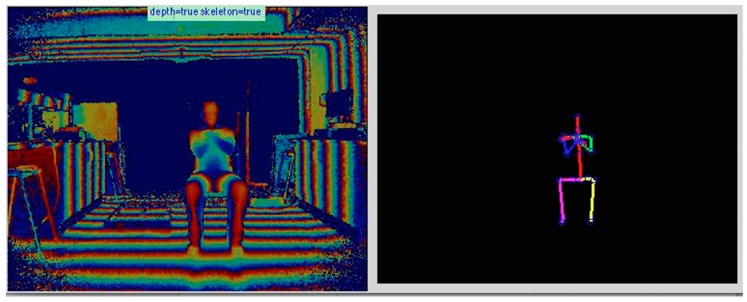
Example output of the Microsoft Kinect One depth sensor and skeleton model renders in MathWorks Matlab (2016B).

Kinect data was captured using a custom application [20] which interfaced with a Microsoft Kinect One to record depth and mocap data at a 30 Hz sampling rate. The output, including depth and the skeleton model with 25 anatomical landmark locations, is shown in Figure 1. The sensor was placed on a tripod at a 70 cm height with a vertical angle of 0°. Room furniture was removed to enable maximum visibility and reduce occlusion, and lighting was standardised via lighting controls.

**Figure 2:**
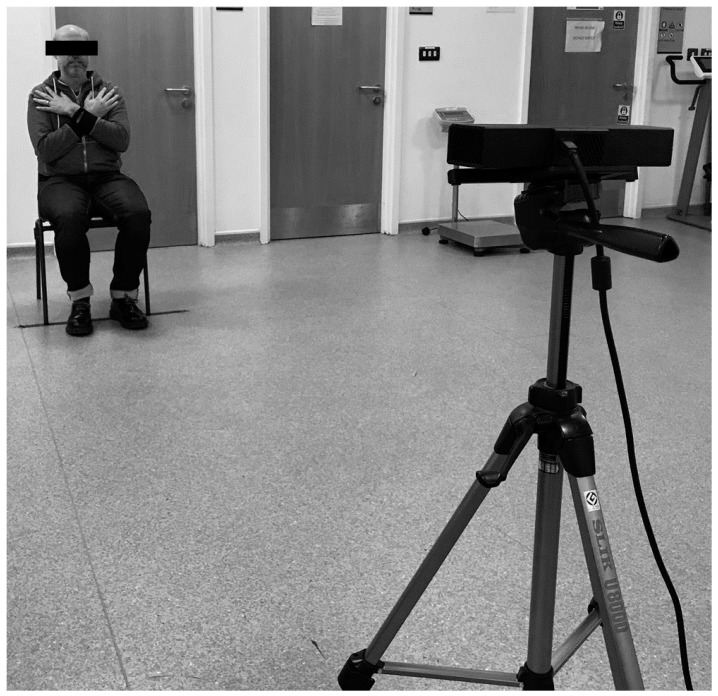
Example recording enviorment with the Microsoft Kinect One placed on a tripod at 70 cm.

Each participant was asked to perform the sit-to-stand starting from a seated position. A chair with a seat height of 44 cm and secure back rest, without arm rests, was used (see Figure 2). When instructed, they had to stand up so that the legs were fully extended, and then sit down again. This was repeated five times with the aim to complete five complete stand/seat cycles within a 60 s period. The arms were held across the chest so that all the power needed to stand and sit was produced by the legs muscles. Each participant was provided with a maximum of three attempts to complete the motion within the time-limit. 

#### 2.1.3. Data Labelling

Two coders were recruited to annotate each motion sequence to identify specific points of interest using a video recording of the session. The following were coded:

1. Peak of each sit-to-stand phase: The coder was asked to locate the minima and maxima of each peak of the standing and sitting repetition.
2. Start and end of each sit-to-stand phase: The coder was asked to identify where they believe the start of the sit-to-stand and end (stand-to-sit) were located.
3. Outlier frames: The K3Da dataset labelled each frame as a ‘good’ frame or ‘outlier’, which was reassessed by coders for agreement.

The locations were recorded for each motion and will be used as ground-truth when comparing the performance of the proposed framework. The inter-coder reliability (±10 frames for each specific point of interest) was calculated at 0.84, and where differences occurred, the coders discussed their differences and came to an agreement (resulting in full agreement). 

### 2.2. Assessment Framework

A single markerless depth sensor was used to record the sit-to-stand motion before the following phases were undertaken in an offline environment.

#### 2.2.1. Phase 1: Outlier Detection

Marker-less mocap systems can occasionally produce unreliable tracking of important anatomical locations (i.e., hand, arms, knees), for instance, an unorthodox body position (i.e., crouching down) or the occlusion of body parts (i.e., one leg hidden behind the other). These recording errors introduce noise in the mocap data, which can impact analysis of the sit-to-stand motion due to subtlety of motion differences [21]. To identify recording errors (outliers), we performed outlier detection based on the Euclidean distance and the principle that mutual Euclidean distances between any joint should not vary with time. A *k*-means clustering algorithm was used to detect outlier (noisy) frames, where mocap data of each frame are clustered into two groups; one which contains the good frames and the other containing the outlier, or poor quality frames. 

A heuristic algorithm was employed to identify the centroid seed for both groups following the proposal of Arthur and Vassilvitskii (2017) [22]. After initialisation, *k*-means clustering is performed to identify and assign a group to the mocap frames. A goodness index is defined, Equation (1), based on the average *L2* norm between the cluster centre (denoted as *c*) and a set of frames (denoted as *F*) assigned to the cluster, as proposed by [23,24]. The cluster which contains the highest *G_c_* value is selected as the *good* cluster, with the remaining cluster being identified as the outlier. This is given as:

(1) $$G_{c}=\frac{\sum_{j=1}^{n}|\left|C_{c}-F_{j}\right|{|}^{2}}{\max\left(n\right)},\;1\;\leq c\;\leq 2.$$

where *n* is the frame index and *j* is the joint index. All frames which lie within the *good* cluster are used for all further analyses, and those labelled as *poor quality* frames are disregarded. 

#### 2.2.2. Phase 2: Feature Generation

**Figure 3:**
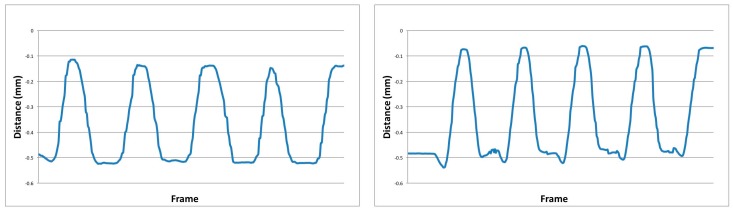
Visual 2-D (*y* axis) representation of the Centre-of-Mass feature encoded from two sequences of sit-to-stand. Left: motion performed by a healthy old participant. Right: motion performed by a young adults.

*Centre-of-Mass (CoM)*: The CoM [25,26] is encoded to describe the anterior-posterior (AP; i.e., forward and backward directional movement) and medio-lateral (ML; i.e., left and right directional movement) directional movement of the participant. Figure 3 demonstrates two-dimensional (2-D) examples of sit-to-stand motion sequences. Let *com* be the encoded CoM derived from three joints (*LeftHip*, *RightHip*, *SpineMid*) given as:

(2) $$com={\left[\bar{x},\;\bar{y},\bar{z}\right]}^{n}=\bar{x}=\frac{\sum_{j=1}^{3}F_{n,x}}{3},\;\bar{y}=\frac{\sum_{j=1}^{3}F_{n,y}}{3},\bar{z}=\frac{\sum_{j=1}^{3}F_{n,z}}{3}.$$

where $\bar{x},\;\bar{y},\bar{z}$ is the derived mean value, *n* is a frame index, and com is a set of mean values.

*Upper Body Flexion Angle (UBFA)*: Previous work has sought to represent the postural body *lean* by encoding the angle of the spine in relation to the floor plane [9,27]. However, implementing this type of computation for motions such as sit-to-stand will not suitably encode the upper body flexion of rising and sitting in a chair. In this work, we propose the Upper Body Flexion Angle (UBFA) to represent the *lean* of motions that require priority use of the upper torso. The UBFA is computed between the *SpineBase* and *Neck*, given as:

(3) $$\theta =arc\cos{\left(\frac{F_{SpineBase}{}^{\circ}F_{Neck}}{\left|\left|F_{SpineBase}\right|\right|\;\left|\left|F_{Neck}\right|\right|\;}\right)}^{n}.$$

*Upper-frame Velocity (UfV)*: The Upper-frame Velocity (UfV) is encoded by computing the temporal difference of the motion in time sequential order between each frame (*n*–*n*_−1_) of the CoM (*com*) feature defined earlier. 

The CoM, UBFA, and UfV are concatenated into single matrices and used for analyses. 

#### 2.2.3. Phase 3: Transition Detection

**Figure 4:**
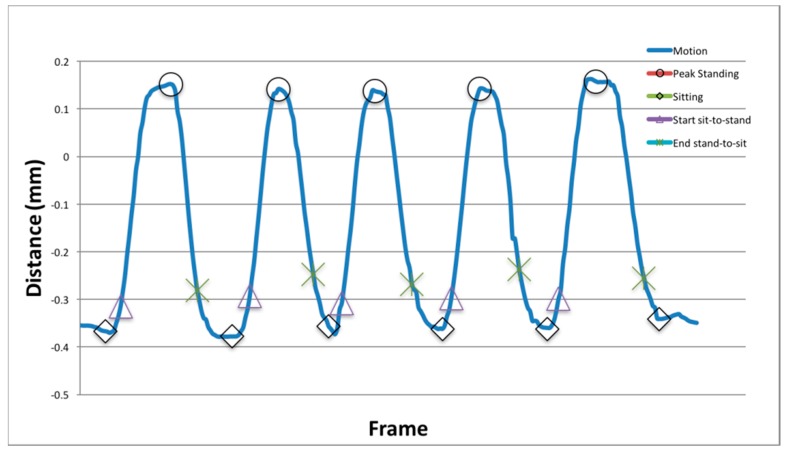
Example output of the proposed transition detection framework derived from the Centre-of-Mass feature vector.

The vertical displacement (*y-axis*) of the CoM (*com*) feature defined earlier was used to identify the transitions between sit-to-stand and stand-to-sit. Following a similar approach described by Ejupi et al. (2015) [13], an automatic identification technique was applied to identify the following phases: sitting, sit-to-peak-standing transition, and peak-standing-to-sitting transition. The automatic transition detection framework (Figure 4) is defined in the following three-step process:

1. Peak-standing and sitting: The *y-axis* of the CoM (*com*) feature was low-pass filtered using a Butterworth filter with a normalised cut-off frequency of six frames. Peak standing and sitting points were detected using the inverse maxima, to identify the local minima and maxima of the vector.
2. Start and end of each sit-to-stand phase: The start of the standing and sitting phase commenced when the following conditions were met; start of the sit-to-stand motion was defined as the first vertical increase above a threshold value, defined as the vertical mean (plus 15%), and the final stand-to-sit decreasing below the mean (minus 15%).
3. Limitation: The maximum number of completed transitions was set at five, the required number for the sit-to-stand motion, meaning that if a participant performed more than five, they were not used in computation.

The motion was then divided into separate segments, sit-to-stand-to-sit, for analysis. The seated start and end of the sequence is removed and not factored into the analyses. 

### 2.3. Statistical Analyses

Analysis of Microsoft Kinect Data was performed using customised scripts in MathWorks Matlab (2016b) and statistical analyses were undertaken using the statistical software package STATA (version 14.0) with statistical significance defined as a *p* value < 0.05. The AP, ML, UBFA, and UfV Sit/Stand values were presented as absolute values. Comparison between participant data was assed using Repeated Measures ANOVA to measure within-group differences. Where significant condition-by-group interaction was found, separate dependent sample *t*-tests were performed. 

In addition, the following parameters are defined:

1. Stand Time (s): The time taken between each peak-sitting to peak-standing.
2. Sit Time (s): The time taken between each peak-standing to peak-sitting.
3. CoM Stand ML (cm) and AP (cm): The directional movement observed during each peak-sitting to peak-standing.
4. CoM Sit ML (cm) and AP (cm): The directional movement observed during each peak-standing to peak-sitting.
5. Stand UfV (m/s): The velocity observed during each peak-sitting to peak-standing.
6. Sit UfV (m/s): The velocity observed during each peak-standing to peak-sitting.
7. Stand UBFA (deg): The angle of the torso observed during each peak-sitting to peak-standing.
8. Sit UBFA (deg): The angle of the torso observed during each peak-standing to peak-sitting.
9. Total Time (s): The total time is computed from the first peak-sitting to the last peak-sitting (5 repetitions).

## 3. Results

The participants (*n =* 40) are divided into three groups based on their age and self-reported physical performance ability; young adult (*n =* 15), healthy old person (*n =* 10), and masters athletes (*n =* 15). There was a statistical difference between age, height, and body mass index between groups (see Table 1). Each participant typically took no longer than 15 s (450 frames, 30 Hz recording rate) to complete the movement, and no participant required and/or requested a second attempt. 

### 3.1. Outlier Detection

The proposed outlier detection method performed strongly when assessed on the ability to detect individual outlier frames when compared to manual annotation (as annotated in [19]). Overall detection accuracy was 93.87% (±8.84), and each participant group obtained the following results; young adult obtained 96.47% (±2.96), healthy old obtained 87.16 (±12.40), and masters athletes obtained 95.77% (±7.52). The reasons behind misclassification were explored further, and it was found that this was due to occlusion of the body, noisy outer limbs due to inaccurate tracking, and loose clothing causing tracking inaccuracies. 

### 3.2. Transition Detection

**Figure 5:**
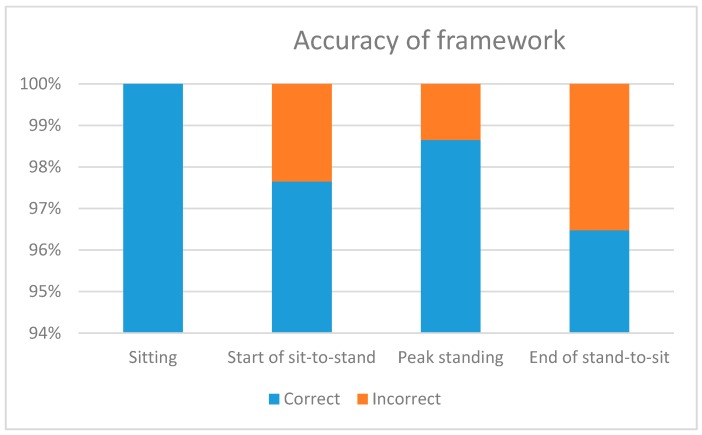
Classification results for each phase. Correct classifications versus incorrect classifications.

Detection of the peak standing point, sitting, start of sit-to-stand, and end of stand-to-sit was compared to manual annotation to compute the accuracy and reliability of detection. Points of interest were determined as correct if they lay within ±10 frames of the manual annotation. The rates for points of interest are presented in Table 2 alongside the accuracy results in Figure 5. Detection rates across the transition phases was high, and the framework can identify the sitting phase; however, there was misclassification between sitting and end of stand-to-sit due to similarities between both phases. 

### 3.3. Identifying Subtle Differences

The framework was able to detect subtle differences between participant groups based on an automated detection of the points of interest (see Table 3); the results are in-line with the literature and indicate the potential use in a healthcare setting [2]. Young adults could complete the sit-to-stand (five repetitions) in less than 8 s compared with healthy old people, who took an average of 12 s. It was observed that for Stand time, Sit UBFA, and UfV, there were significant differences between the young adults and masters athletes. 

## 4. Discussion and Conclusions

In this work, we utilise a depth sensor and automated framework to identify a range of clinically relevant outcome features that may be useful to a clinician in providing greater insight into the performance capability of a participant. Unique insights were obtained for each group. Young adults could execute the sit-to-stand, but presented large AP and ML. The healthy old group were able to execute the sit-to-stand, but presented a reduced AP and ML sway and an increase in time taken to stand and sit. Masters athletes could execute the sit-to-stand with relative ease, with little impediment to their motion, but presented reduced upper body lean when standing and sitting. 

Comparing the performance between participant groups demonstrates the ability of the system to distinguish between effects of ageing. The young adults could perform the sit-to-stand with little impediment to their motion and were able to maintain control. While there is no doubt that masters athletes maintain a high physical capability [28], performance nevertheless declines with advancing age alongside loss of muscle power and cardiopulmonary function [29,30,31], so it is possible that the balance and performance of movements such as sit-to-stand in healthy old people and masters athletes decline with increasing age and loss of muscular control. 

This work exclusively focused on the use of depth sensor technology due to its ability to track human motion without any physical anatomical landmarks, sensors, or devices being placed on the participant’s body. There are several studies that have utilised wearable technology (i.e., mobile devices, accelerometer, and gravity sensors) to track balance and sit-to-stand motions successfully; however, they are only capable of providing outcomes in relation to where the device/marker has been located, which means that the body itself is not being assessed, and they are expensive to implement widely [32,33,34]. However, future work should explore uniting both modalities to provide a holistic overview of the execution of balance and sit-to-stand motions. 

There are several limitations in this study, most of which relate to the use of technology in making a clinical judgement. First, the Kinect is sensitive to light, occlusion, and placement, which could impact the tracking of the skeletal joints and the outcomes from the framework. Future studies are needed to improve tracking in different environments. Second, this study relied on labelling annotated by human coders, and there is a potential that bias may impact coding. Future studies are needed to explore the relationship between human coding and computerised coding comparisons. Third, the detection of outlier frames may have impacted the detection of phases. Future work should seek to explore the use and reliability of interpolation methods, such as [35], to replace outlier frames with an estimation of the correct frame. Finally, this study presented multiple analyses; however, we should consider how a clinician would interpret these results. Future work should seek to explore how we should present data in a clinical context. 

We have proposed a framework which unites depth sensor technology and feature extraction to assess the sit-to-stand motion sequence. The framework has been shown to be reliable and accurate in evaluating the transition phases and providing clinical outcome measures. Future work will focus on future clinical validation, increase the number of participants, improve reliability, and extend the framework to analyse a wide range of motions. 

## Figures and Tables

**Table 1:** Characteristics of participants divided by group.

Parameter (SD)	Young Adult	Healthy Old	Masters Athletes	*p*-Value
Age, years	26.40 (±3.16)	74.90 (±4.11)	66.93 (±5.03) ^a,b^	0.00
Height, cm	176.47 (±8.59)	170.30 (±5.97) ^c^	166.01 (±10.07)	0.04
Weight, kg	77.93 (±18.11)	80.25 (±15.32)	61.90 (±9.39)	0.594
Body mass index	23.01 (±5.70) ^b,c^	22.65 (±5.38)	19.14 (±2.11)	0.04

The *p*-value represents the main effect obtained from the ANOVA. Results from dependent comparisons are included as ^a^ significantly different from Young; ^b^ significantly different from healthy old; ^c^ significantly different from masters athletes.

**Table 2:** Transition detection rates for each point of interest.

Parameter	Average Detection Rate (SD)
Sitting	6 (±0)
Start of sit-to-stand	4.76 (±0.48)
Peak standing	4.93 (±0.35)
End of stand-to-sit	4.34 (±0.63)

**Table 3:** Computed results for the sit-to-stand motion for each participant group.

Parameter	Young Adults	Healthy Old	Masters Athletes	*p*-Value
Stand Time (s)	1.02 (±0.18)	2.02 (±0.21)	1.51 (±0.19) ^a^	0.02
CoM Stand ML (cm)	0.24 (0.05)	0.03 (0.26)	0.17 (0.06)	0.56
CoM Stand AP (cm)	0.21 (±0.01) ^b^	0.01 (±0.19)	0.04 (±0.14)	0.04
Stand UBFA (deg)	12 (±2.86)	18 (±4.09)	14 (±3.58)	0.62
Stand UfV (m/s)	0.82 (±0.19)	0.71 (±0.38)	0.73 (±0.19)	0.16
Sit Time (s)	0.92 (±0.23)	1.47 (±0.73)	0.98 (±0.35)	0.23
CoM Sit ML (cm)	0.22 (0.06)	0.04 (0.28) ^a,c^	0.22 (0.09)	0.00
CoM Sit AP (cm)	0.22 (±0.03)	0.03 (±0.17)	0.05 (±0.16)	0.53
Sit UBFA (deg)	17 (±3.19)	16 (±3.71) ^a^	10 (±2.38) ^a^	0.05
Sit UfV (m/s)	0.98 (±0.19)	0.78 (±0.58)	0.83 (±0.21) ^a^	0.04
Total time (s)	7.98 (±2.09)	12.18 (±3.76)	9.28 (±0.94)	0.24

The *p*-value represents the main effect obtained from the ANOVA. Results from dependent comparisons are included as a significantly different from Young; b significantly different from healthy old; c significantly different from masters athletes.
